# Differential Natural Selection of Human Zinc Transporter Genes between African and Non-African Populations

**DOI:** 10.1038/srep09658

**Published:** 2015-04-30

**Authors:** Chao Zhang, Jing Li, Lei Tian, Dongsheng Lu, Kai Yuan, Yuan Yuan, Shuhua Xu

**Affiliations:** 1 Chinese Academy of Sciences (CAS) Key Laboratory of Computational Biology, Max Planck Independent Research Group on Population Genomics, CAS-MPG Partner Institute for Computational Biology (PICB), Shanghai Institutes for Biological Sciences, Chinese Academy of Sciences, Shanghai 200031, China; 2 School of Life Science and Technology, ShanghaiTec University, Shanghai 200031, China; 3 Collaborative Innovation Center of Genetics and Development, Shanghai 200438, China

## Abstract

Zinc transporters play important roles in all eukaryotes by maintaining the rational zinc concentration in cells. However, the diversity of zinc transporter genes (ZTGs) remains poorly studied. Here, we investigated the genetic diversity of 24 human ZTGs based on the 1000 Genomes data. Some ZTGs show small population differences, such as *SLC30A6* with a weighted-average *F_ST_* (WA-*F_ST_* = 0.015), while other ZTGs exhibit considerably large population differences, such as *SLC30A9* (WA-*F_ST_* = 0.284). Overall, ZTGs harbor many more highly population-differentiated variants compared with random genes. Intriguingly, we found that *SLC30A9* was underlying natural selection in both East Asians (EAS) and Africans (AFR) but in different directions. Notably, a non-synonymous variant (rs1047626) in *SLC30A9* is almost fixed with 96.4% A in EAS and 92% G in AFR, respectively. Consequently, there are two different functional haplotypes exhibiting dominant abundance in AFR and EAS, respectively. Furthermore, a strong correlation was observed between the haplotype frequencies of *SLC30A9* and distributions of zinc contents in soils or crops. We speculate that the genetic differentiation of ZTGs could directly contribute to population heterogeneity in zinc transporting capabilities and local adaptations of human populations in regard to the local zinc state or diets, which have both evolutionary and medical implications.

Zinc (Zn), an essential trace mineral, is required for the structures and functions of many proteins, including enzymes and transcription factors, and is critical for their biological activities, such as cellular metabolism and gene expression^1^,^2^. Previous studies have estimated that >3% or even as much as 10% of human proteins are zinc-binding proteins^3^, indicating that the maintainability of cellular zinc concentrations and distribution in human cell compartments is of extreme importance. Generally, there are 24 zinc transporter genes (ZTGs) involved in cellular zinc homeostasis in the human genome^4^,^5^ among which 10 genes of *SLC30A* family lower intracellular cytoplasmic zinc by mediating zinc efflux from cells or influx into intracellular vesicles^6^, while 14 genes of *SLC39A* family transporters mobilize zinc in the opposite direction^7^.

Because of the fundamental roles in biological processes, mutations in ZTGs are likely to break the balance of zinc in cell compartments, resulting in improper biological functions of zinc-dependent proteins and subsequent severe diseases or impaired development. For instance, studies have shown that acrodermatitis enteropathica (OMIM 201100) was caused by the loss of function of one of or both *SLC39A4* alleles, leading to a diminished uptake of dietary zinc, increased sensitivity to zinc deficiency, and severe growth retardation^8^. Some reports suggested that changes in intercellular zinc level are associated with cancer progression as the expression of zinc transporters in many cancers altered. For example, increased expression of ZIP6, ZIP7 and ZIP10 have been observed to contribute to zinc hyper-accumulation in breast tissue and breast cancer^9^. Other investigations also indicated that ZIP6 and ZIP10 play critical roles in breast cancer progression^10^,^11^. Similarly, elevated ZIP4 expression level in pancreatic cell is associated with pancreatic cancer^12^,^13^,^14^,^15^. Besides cancers, zinc transporters are involved in some complex diseases. Sladek *et al.* found that rs13266634, a non-synonymous SNP (R325W) in *SLC30A8* is related to type 2 diabetes^16^. *SLC30A8* encodes a zinc transporter which is expressed solely in secretory vesicles of β-cells and the overexpression of *SLC30A8* in insulinoma cells increases glucose-simulated insulin secretion^17^. Another example is that *SLC39A8* was observed to have relationship with body mass index^18^. Such disruptive mutations that reduce fitness would be removed from population by purified selection.

On the other hand, functionally important ZTGs may also be subjected to positive selection for acquired changes that increase fitness. Recently, Engelken and Carnero *et al.* reported that the human intestinal zinc uptake transporter, *SLC39A4*, has undergone positive selection in Sub-Saharan African populations^19^. By carrying out cell transfection assays of putative functional variants, they validated an amino acid change (L372V) in *SLC39A4* which was shown to lead to reduced zinc uptake in the African isoform. According to previous studies, zinc contents in soils or crops are extremely diverse across continents or countries and it is estimated that African populations have undergone severe zinc deficiency, according to various kinds of zinc deficiency indicators^20^. We hypothesize that, due to the uneven global distribution of absorbable zinc in soils, crops and different diet habits, some ZTGs with adaptable variations in different populations might be underlying natural selection as their transporting capability changed, thus maintaining the balance of intercellular or serous zinc in the human body.

We, therefore, systematically analyzed the patterns of genetic diversity and signals of natural selection for 24 14ZTGs in 14 worldwide populations. In this study, we showed that ZTGs harbor many more highly population-differentiated variants compared with random genes and discussed the potential underlying forces shaping the genetic diversity of ZTGs. Further, we reported that *SLC30A9* was underlying natural selection in both East Asians (EAS) and Africans (AFR) but in different directions. By performing a correlation, we found that the evolutionary force underlying the selective sweep of *SLC30A9* may be the uneven worldwide zinc distribution in soils or corps. Moreover, we predicted 17 potentially functional SNPs, which may guide the study of molecular mechanism of ZTGs. Our results may subsequently increase our understanding of the evolutionary forces that affect ZTGs, as well as augmenting our knowledge of gene function on zinc homeostasis in different populations and the mechanisms of zinc-related diseases.

## Results

### Genetic differentiation of ZTGs among populations

We first performed an analysis of molecular variance (AMOVA) to examine whether genetic variance among four continental regions is significantly different from populations within each region (Table S1 and Figure S1). Our analysis showed that *SLC30A9* and *SLC30A3* had a greater proportion of variance among continental groups than within group (empirical P < 0.05), indicating that the two genes were genetically differentiated among continental populations. On the contrary, *SLC30A6* showed a much lower proportion of variance between groups compared with most of the other genes genome-wide (empirical P < 0.05), suggesting that *SLC30A6* may be functionally conserved.

We next calculated a weighted-average *F_ST_* (referred to as WA-*F_ST_*) for each gene. WA-*F_ST_* employs a weighted average of the *F_ST_* values calculated from variants in a gene or a genomic region. Among the 24 14ZTGs, *SLC30A9* and *SLC30A3* showed high WA-*F_ST_* values (> = 0.19, top 5% cutoff of the whole genome), indicating that the variation composition is substantially different between populations. Therefore, the functional variants associated with specific haplotypes are also distributed heterogeneously across populations. On the contrary, *SLC30A6* with a low WA-*F_ST_* (< = 0.128, empirical P < 0.05) may have limited genetic heterogeneity across populations (Table S1 and Figure S2).

**Figure 1:**
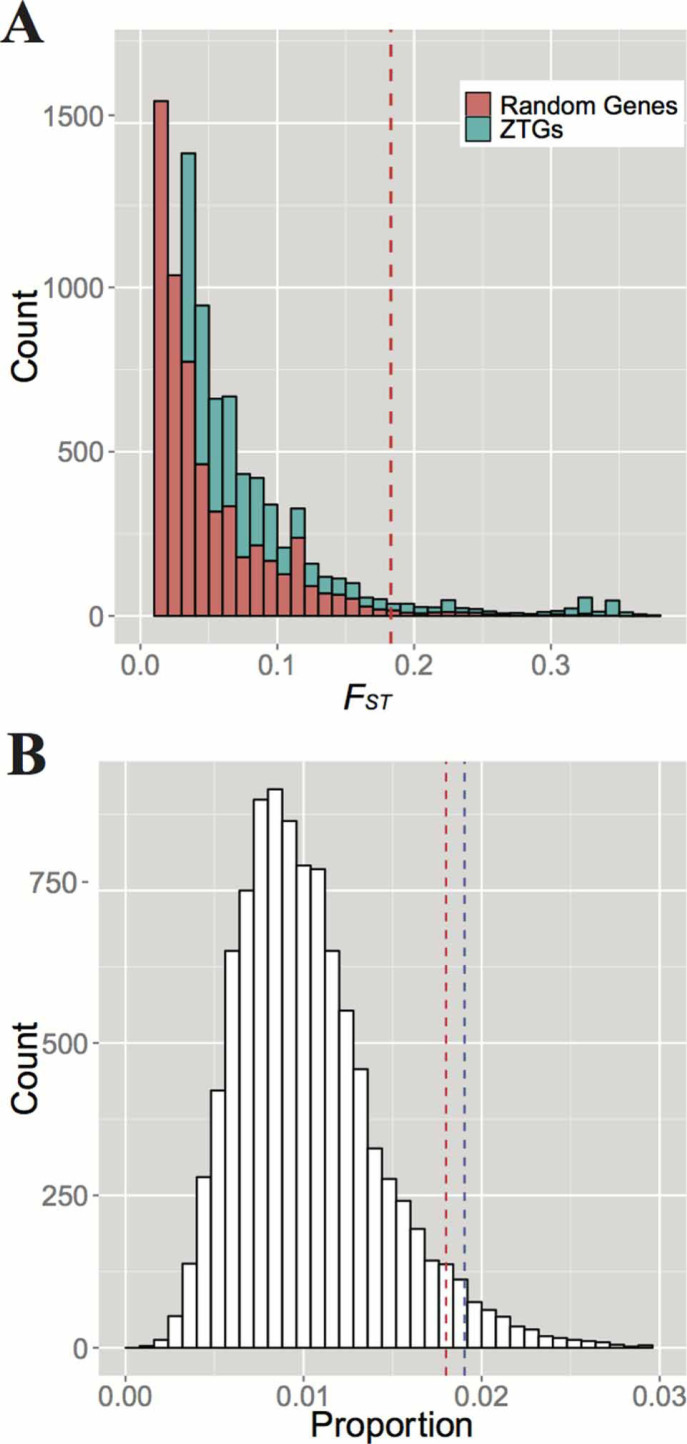
ZTGs show a higher proportion of locus-specific *F_ST_* when compared to random genes. (A) The locus-specific *F_ST_* distribution of all sites located in 24 14ZTGs and 24 random genes. In the figure, the dashed line represents the cutoff with the empirical P-value of 0.01, i.e. *F_ST_* = 0.183, the highest 1% of the genome-wide locus-specific *F_ST_*. (B) Distribution of a 10,000 times permutation for the proportion of high *F_ST_* in 24 random genes. The red dashed line represents the cutoff with the empirical P-value of 0.05, i.e., proportion = 0.018, while the blue line represents the proportion of loci with high *F_ST_* in ZTGs (0.0182).

We then calculated the unbiased locus-specific *F_ST_*, following Weir and Hill^21^ in determining the highly differentiated loci in ZTGs (see Materials and Methods). A locus-specific *F_ST_* measures the apportionment of genetic variation of one specific SNP between populations. A high locus-specific *F_ST_* value indicates that the corresponding allele is substantially different between populations, while SNPs with low locus-specific *F_ST_* have small genetic difference and little functional heterogeneity across populations. The locus-specific *F_ST_* values varied widely among SNPs in ZTGs, with the maximum *F_ST_* for SNP rs1871534 in *SLC39A4* at 0.763 and the average value at 0.018. We used the highest 1% of the genome-wide locus-specific *F_ST_* (0.183) as the cutoff for a significant signal of population differentiation. As a result, compared to the *F_ST_* distributions of 24 random genes, ZTGs showed significantly higher percentages of variants with high *F_ST_* and average *F_ST_* values (the mean *F_ST_* value for random genes is 0.015). In total, there were 361 (of 19888) (1.82%) SNPs in ZTGs with *F_ST_* > = 0.183, while the percentage was only 0.56% (131 of 23,237) in random genes (Figure 1A). In particular, 17% SNPs in *SLC30A9*, 8% SNPs in *SLC30A3* and 6% SNPs in *SLC30A4* harbored genetic variants with significantly high *F_ST_*. The proportions in *SLC30A1*, *SLC30A10*, *SLC30A5* and *SLC30A6* were relatively lower than the other genes (Figure S3). Finally, a permutation test (see Materials and Methods) (P = 0.048) was applied to exclude the possibility that the population differentiations in ZTGs resulted from sampling effect (Figure 1B). When we used the top 5% of the genome-wide locus-specific *F_ST_* as the cutoff for a significant signal of population differentiations, the P-value (<0.01) was also significant (see next section for more details). All these results suggested that the differentiations of ZTGs among populations were unlikely to be caused by any stochastic factors.

### Population differentiations and potential effects of functional SNPs

The above analyses focused on general patterns of *F_ST_* distribution of all loci in ZTGs. However, the genetic variants that directly affect protein functions and gene expressions are functional SNPs, which are more likely to have true associations with zinc transporting or diseases. In our study, SNPs that (i) change the amino acid sequence of protein (non-synonymous SNPs, nSNPs), (ii) correlate with gene expression (eQTL (expression quantitative trait loci) SNPs), (iii) alter splicing (splicing SNPs) and (iv) are associated with diseases (in the GWAS catalog or other databases) were investigated (see Materials and Methods).

**Figure 2:**
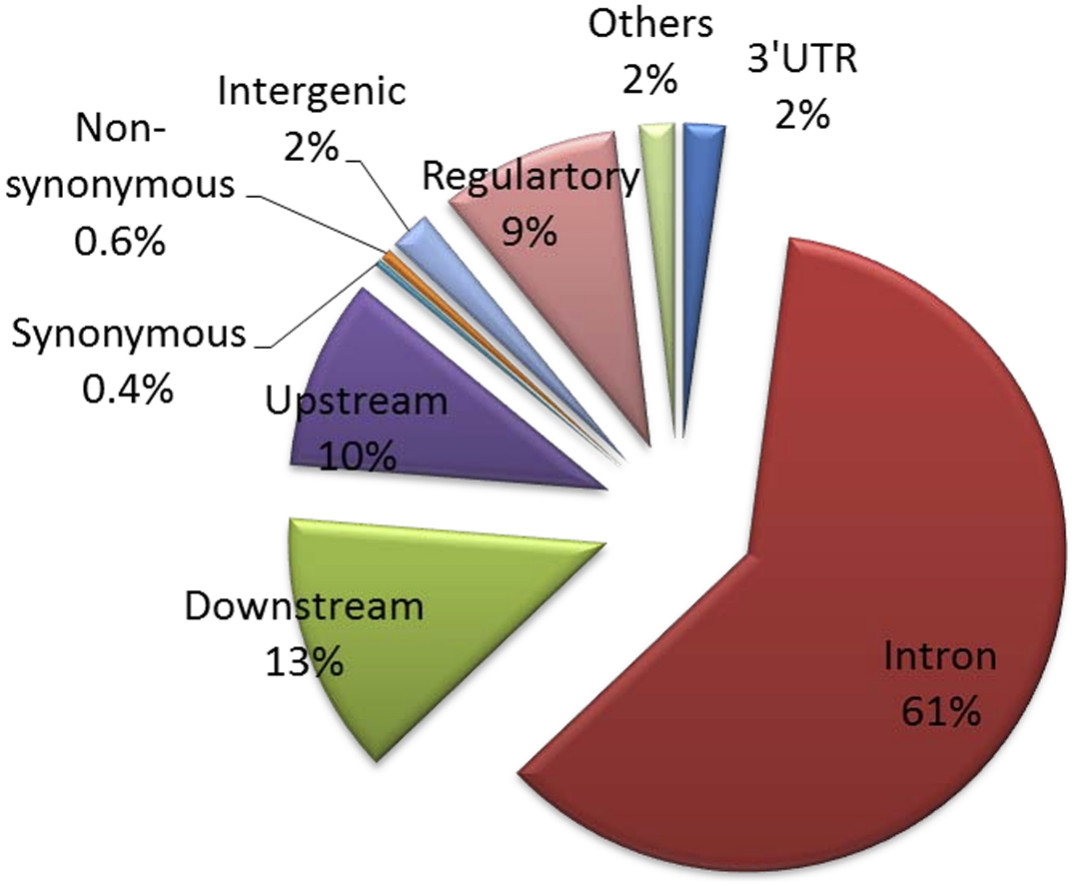
The variant effect prediction of loci in ZTGs, including up- and down-stream 10-kb regions.

Compared to the *F_ST_* distributions of random genes, the ZTGs showed significantly higher percentages of variants with high *F_ST_*; i.e. there were 932 out of a total 19,888 14SNPs with *F_ST_* > = 0.092 (the top 5% *F_ST_* cutoff) (P < 0.01, 10,000 permutations). We first predicted the functional types of all 19,888 14SNPs in ZTGs and 5,728 14SNPs located within the upstream and downstream 10 14Kb of ZTGs by consulting the variance effect prediction tools from the Ensembl website (Figure 2). As shown in Figure 2, 61% SNPs were located in the intronic region, 2% SNPs in the intergenic region, 13% SNPs in the downstream region and 10% SNPs in the upstream region, respectively. In particular, 195 14nSNPs and 47 splicing SNPs were observed. Furthermore, we found an additional 25 14eQTL SNPs in RegulomeDB, 16 14SNPs in the GWAS catalog, and 3 14SNPs in the GKB database. From those 25,616 14SNPs, we screened 17 functional SNPs with *F_ST_* > = 0.092, including 7 14nSNPs, 1 splicing SNP, 6 14eQTL SNPs and 3 disease-related SNPs (2 in the GWAS catalog and 1 in GKB) (Table 1).

All of the 17 functional SNPs showed high *F_ST_* values across populations and therefore were assumed to have a higher potential functional impact on zinc transporting or were associated with diseases. Non-synonymous SNPs can cause a structural change in the protein product, which potentially leads to a minor or major phenotypic change. We applied Polyphen-2^22^ to predict how amino acid variants might change the function of ZTGs peptides (Table 1). Interestingly, rs1871534 was predicted to be potentially damaging, with a score of 0.991; it was located in the second transmembrane domain (TMD) in *SLC39A4*, predicted by HMMTOP (Table 1 and Figure S4) and Engelkin *et al.* have proved that it affects zinc transporting ability of *SLC39A4* in in vitro experiments^23^. Besides nSNPs, other types of SNPs may also be important. One splicing SNP (rs61756712), 6 eQTL SNPs (rs759071, rs6832846, rs151368, rs11889699, rs17278473 and rs151372) and 1 regulatory region variant (rs17060812) may both contribute to gene expression and thus alter intercellular zinc levels. Moreover, two SNPs included in GWAS catalog were identified. For example, rs950027 in *SLC30A4* was reported to be related with response to fenofibrate^24^, and rs11264736 in *SLC39A1* was associated with lentiform nucleus volume, which can lead to disorders, including Parkinson's disease^25^. Therefore, the 17 highly differential functional SNPs could contribute to population heterogeneity in zinc transporting capability, providing guidance for studying the transporting mechanism of ZTGs at a molecular level.

### Frequency distributions of functional SNPs among different populations

**Figure 3:**
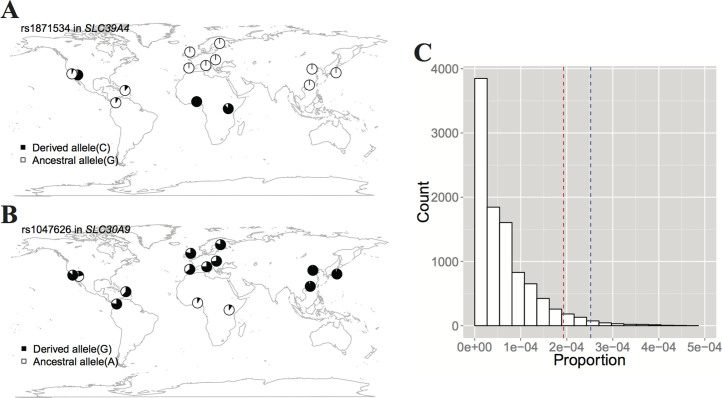
Worldwide derived allele frequency (DAF) distributions of highly differential nSNPs. (A) DAF of rs1871534 in *SLC39A4*, which is nearly fixed in Africans but almost absent in non-Africans. (B) DAF of rs1047626 in *SLC30A9*. This SNP shows a pattern opposite of rs1871534. (C) 10,000 times permutation for the proportion of nSNPs with a high *F_ST_* (> = 0.092) and a low DAF (<0.05) in CEU and CHB in random genes. The red and blue dashed lines represent the cutoff with the empirical p value 0.05 and a proportion in ZTGs, respectively. nSNPs with a high *F_ST_* and a low DAF in CEU and CHB are significantly enriched in ZTGs (P = 0.019), which is possibly caused by natural selection. World maps here were created by R packages (http://www.r-project.org/).

We further analyzed the distribution of allele frequencies of candidate SNPs in different populations worldwide (Table 1). Interestingly, the 7 14nSNPs in the 17 candidate SNPs had two distinct patterns. One is that the derived allele frequencies (DAF) of 5 14nSNPs (rs1871534, rs2466517, rs11011935, rs2010519 and rs75920625) were much higher in Africans compared with non-Africans in whom the DAF were close to 0 (Figures 3A and S5A–D). For instance, the DAF of rs1871534 was 0.006, 0.000 and 0.983 in CEU, CHB and YRI, respectively. The other pattern showing completely opposite trends was that rs1047626 and rs2272662 had high DAF in Europeans and East Asians but the derived alleles of the two SNPs were nearly absent in Africans (Figures 3B and S5E). In particular, the DAF of rs1047626 in *SLC30A9* was amost 1 in East Asians (CHB: 0.964) but was 0.080 in YRI (Table 1). In spite of the different patterns, they showed the same trends indicating Africans and non-Africans were different in these nSNPs. A 10,000 permutations was implemented to validate that nSNPs with high *F_ST_* (> = 0.092, the top 5% *F_ST_* cutoff), and high DAF in YRI but low DAF (<0.05) in CEU and CHB were significantly enriched in ZTGs (P = 0.019) (Figure 3C). Therefore, it is unlikely the specific pattern of these candidate SNPs have been caused by genetic drift, possibly resulting from natural selection instead.

### Detecting regional natural selection in ZTGs

**Figure 4:**
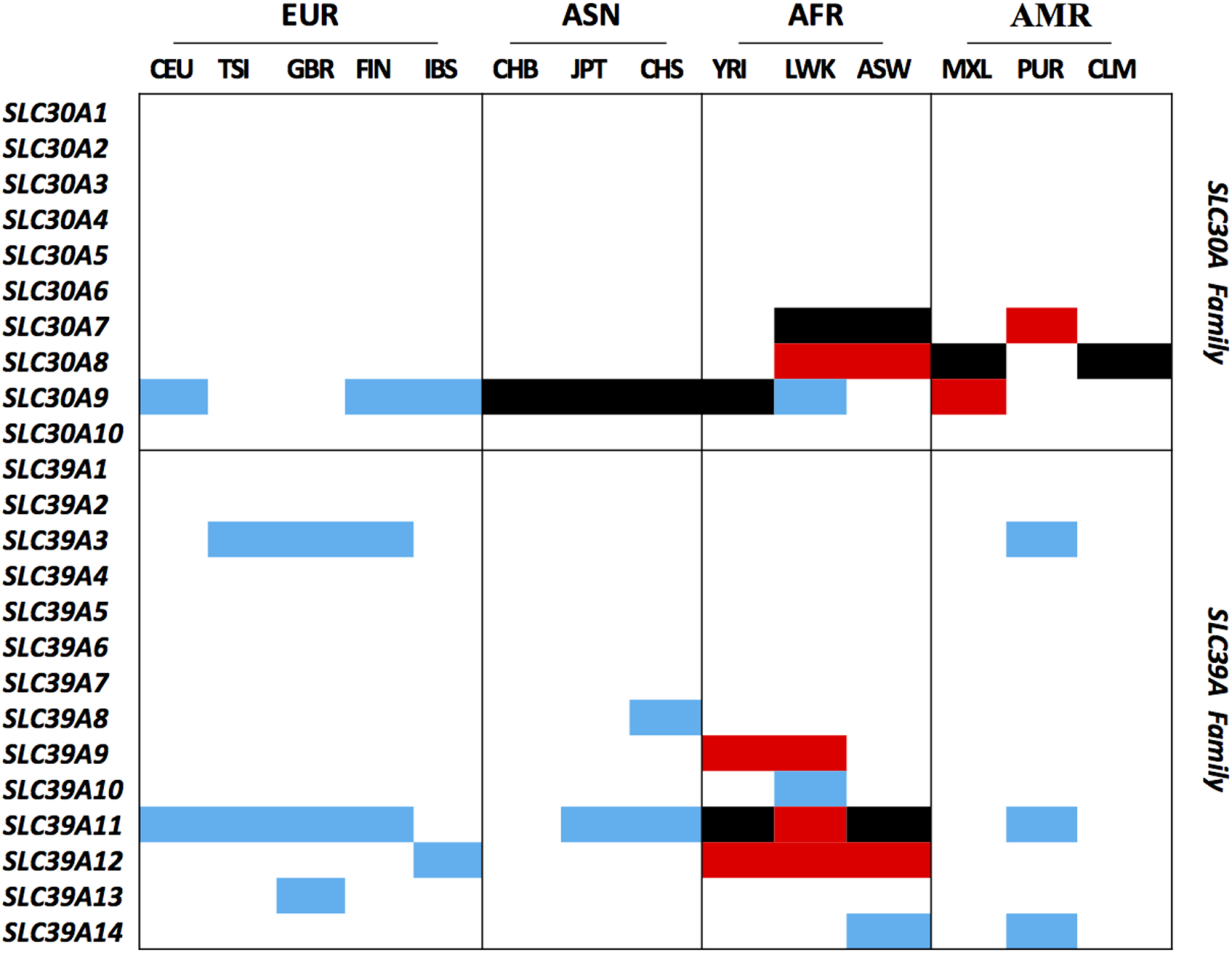
Global natural selection pattern for ZTGs. The blue blocks indicate the significant signals found by the iHS test; the red color indicates the significant signals found by Sweep Finder, and black indicates the overlapping signals for both methods.

The AMOVA and *F_ST_* analyses revealed overall patterns of ZTGs genetic differentiations among worldwide populations. We then applied two commonly used statistics, the integrated haplotype score (iHS) and the composite likelihood ratio (CLR) (see Materials and Methods), to detect the signals of selection in the ZTGs. We found ZTGs exhibiting evidence of recent positive selection. There was a certain consensus between the results of the iHS and CLR tests, which supported the reliability of the analysis (Figure 4). Figure 4 illustrates the signals of positive selections for ZTGs in different populations. The iHS test supported the indication of selection on several genes. *SLC30A7* exhibited signals in two African populations (LWK and ASW) (Figures 4 and S6). *SLC30A8* appeared to have been selected in primarily American populations (MXL and CLM) (Figures 4 and S7). *SLC30A9* underwent selection, as indicated by iHS, in all of the Asian populations, three European populations (CEU, FIN and IBS) and two African populations (YRI and LWK) (Figures 4 and S8). All populations shared the iHS signals in *SLC39A11* except IBS, CHB, LWK, MXL and CLM (Figures 4 and S9). There were 6 genes (*SLC30A7*, *SLC30A8*, *SLC30A9*, *SLC39A9*, *SLC39A11* and *SLC39A12*) exhibiting significant signals in the CLR test. For instance, *SLC39A9* shows strong selection signals in YRI and LWK. Four genes (*SLC30A7, SLC30A8, SLC30A9* and *SLC39A11*) showed overlapping signals in both methods in some populations. For example, *SLC30A7* harbored both iHS and CLR signals in LWK and ASW with *SLC30A8* in MXL and CLM. *SLC39A11* had overlapping signals for both methods in YRI and ASW. Specifically, a previous study reported that *SLC30A9* was strongly selected in East Asian populations, and it is among the top 10 selection signals in the genome-wide detection of positive selection in human populations^26^. However, we found that *SLC30A9* underwent selection in different continental groups, including Africans and East Asians using both iHS and CLR tests (Figures 4 and S8), suggesting that the selective signals *in SLC30A9* were very strong.

### Directional selections acted on *SLC30A9* differently in Africans and East Asians

**Figure 5:**
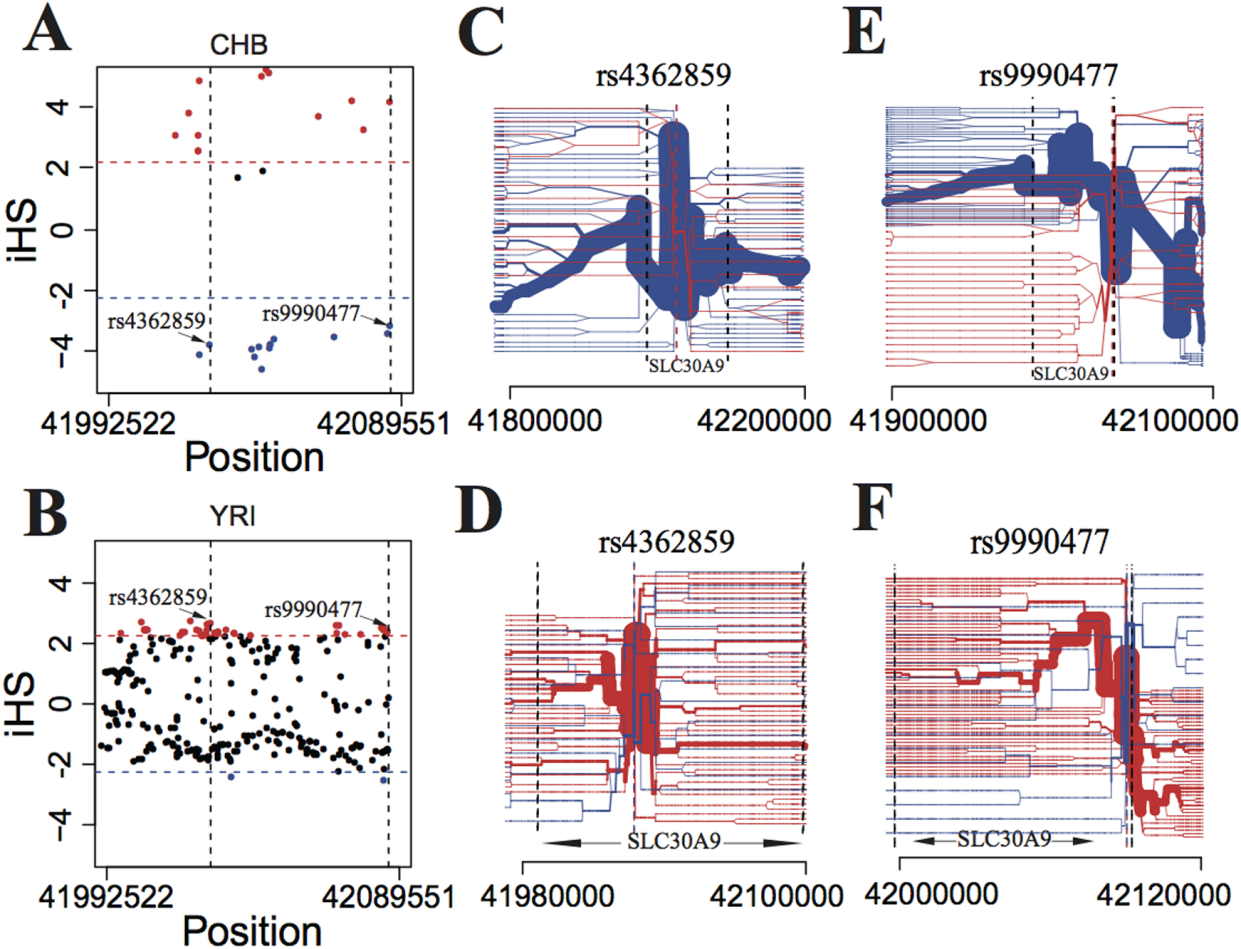
*SLC30A9* shows selective signatures in both Africans and East Asians. (A), (B) Plots of iHS values for SNPs of *SLC30A9* in CHB (A) and YRI (B). The values of standardized iHS for sNPS with minor allele frequency >5% are plotted against the the genomic positions (hg19). The 95% cutoff value of the empirical distribution of standardized iHS is given in each plot (dashed line). Red and blue dots representing ancestral and derived alleles of SNPs were selected, respectively. Only rs4362859 and rs9990477 harbor significant but opposite iHS values in both CHB and YRI. (C), (D) Haplotype bifurcation diagrams for the core haplotype carrying rs4362859 at *SLC30A9* in CHB (C) and YRI (D). In CHB, the haplotype carrying the derived allele of rs4362859 (blue color) has long-range homozygosity that is unusual given their frequency, while the haplotype carrying the ancestral allele of rs4362859 (red color) is favored. (E), (F) Haplotype bifurcation diagrams for core haplotype carrying rs9990477 at *SLC30A9* in CHB (E) and YRI (F). The pattern of rs9990477 is similar with that of rs4362859.

We further analyzed the selected haplotypes of *SLC30A9* in each population and found that the haplotype favored in the African population was different from that found in East Asians and Europeans (Figure 5). The iHS test generally takes SNPs with minor allele frequency (MAF) large than 5% into consideration. Therefore, there were only 28 and 294 (of 1294) loci with iHS values in CHB and YRI, respectively, leaving numerous numbers of alleles in *SLC30A9* which were nearly fixed and thus could not be estimated. The SNP with a large positive iHS value indicates that the ancestral allele of this respective site hitchhiked with the selected locus, while the SNP with a large negative iHS value indicates the derived allele was favored by selection. According to the iHS distribution of *SLC30A9* in CHB, there was a high proportion of selected SNPs (SNPs with a significant iHS value, P < 0.05) with large negative values (13 of 26) (Figure 5A), while in YRI most of the selected SNPs harbored large positive iHS values (53 of 55) (Figure 5B). That is, the selected haplotype in CHB included approximately 50% of the derived alleles, while merely 3.6% (2 of 55) of selected SNPs hitchhiked with the extended haplotype in YRI, indicating that the haplotypes selected in these two populations were probably different. Moreover, not only were there differences in the ratios of the derived and ancestral alleles selected in CHB and YRI, the SNPs with significant iHS values were unmatched in each population. There were only two selected SNPs (rs4362859 and rs9990477) that were shared by the extended haplotypes in CHB and YRI (Figures 5A–B and S10A–B). For these two SNPs in CHB and YRI, haplotype bifurcation diagrams were drawn^27^, which allow a better understanding of the origin of an observed footprint of selective sweeps (Figure 5C–E). As shown in Figure 5C and E, when the haplotype of *SLC30A9* harbored the derived alleles of rs4362859 (T) and rs28620429 (C) (blue color), it had long-range haplotype homozygosity, which was unusual given their frequency in CHB. However, if the haplotype included ancestral alleles (red color), it extended less compared with the comprised derived ones in CHB. However, the trend in YRI was completely the opposite. That is, the extended haplotype in YRI comprised the ancestral allele of rs4362859 (C) and rs28620429 (T), while the haplotype that included derived alleles had short-range homozygosity (Figure 5D and F). Therefore, according to the differences in the ratios of derived and ancestral alleles selected in CHB and YRI, as well as the unmatched selected SNPs in each population, we inferred that the selected haplotypes in YRI and CHB were different.

**Figure 6:**
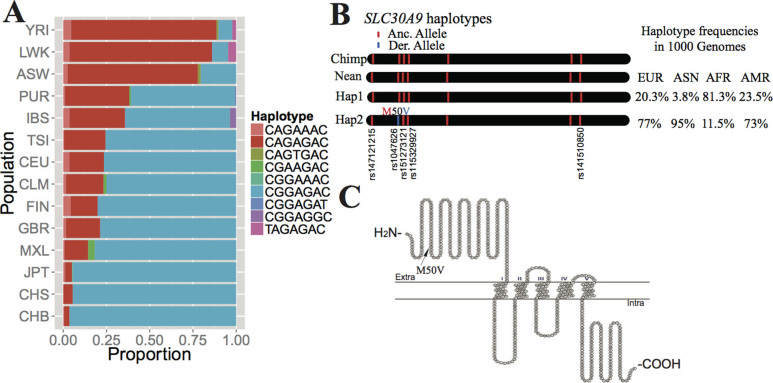
Analysis of putative selected haplotypes carrying 7 14nSNPs in *SLC30A9*. (A) Haplotypes shared among different continental groups. African populations are more likely to harbor haplotype CAGAGAC, while in non-African populations, haplotype CGGAGAC is much more pervasive, especially in East Asians in whom the haplotype frequency is nearly fixed. (B) Graphical depictions of *SLC30A9* haplotypes constructed from 7 14nSNPs with haplotype frequencies derived from the 1000 Genomes Project. Hap1 (CAGAGAC) is the same as the haplotype in a chimpanzee (Chimp) and a Neanderthal (Nean). Hap2 (CGGAGAC) is different from Hap1 at rs1047626 of which the derived allele can alter an amino acid change from methionine acid to valine (M50V). (C) Predicted membrane topology of human *SLC30A9* generated using HMMTOP and visualized with TeXtopo. Location of rs1047626 carried by the possible selected haplotype is indicated.

Next, we investigated the haplotype abundance distribution for *SLC30A9* in each population. Non-synonymous SNPs that may cause a structural change in protein can potentially lead to a minor or major phenotypic change. We observed that 9 different kinds of haplotypes of *SLC30A9* were composed of 7 14nSNPs (Table S2): rs147121215, rs1047626, rs151273121, rs115329927, rs2581423, rs181235146 and rs141510850. There were two main haplotypes that constituted more than 90% of the haplotypes for every population (Figure 6A and Table S2). Strikingly, the haplotype particularly enriched in CHB was CGGAGAC (Haplotype 2, H2) (96.4%), which was nearly fixed. The frequency of H2 in CEU and YRI was 76.5% and 0.08%, respectively (Figure 6A and Table S2), while CAGAGAC (Haplotype 1, H1) was the most common haplotype in African populations (84.1% in YRI). Comparing H1 with H2, we found that the only difference was that H2 contained the derived allele in rs1047626, while H1 had the ancestral one (Figures 6B and S11). The rs1047626 is an nSNP with a high *F_ST_* value (0.387) (Table 1), changing the 50th amino acid in *SLC30A9* from methionine acid to valine (M50V) (Figure 6C). Previous studies also screened a selection signal at this locus^26^,^28^, indicating that it has a high probability to be the causal SNP for the selection signal in East Asia.

### The correlation between haplotype frequencies and zinc deficiency

**Figure 7:**
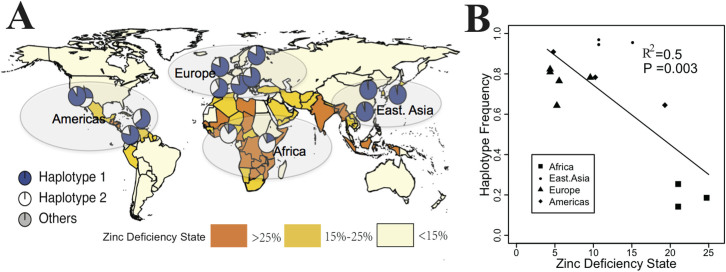
Correlation between zinc deficiency state and frequency of putative selected haplotype. (A) Estimated country-specific prevalence of zinc deficiency and worldwide haplotype frequency distribution for *SLC30A9*. Population zinc deficiency data are based on the composite nutrient composition database, IZINCG physiological requirements, the Miller Equation to estimate zinc absorption and an assumed 25% inter-individual variation in zinc intake (Ref. 20, see Table S3). (B) Correlation test for the zinc deficiency and frequency of Hap2. World map was created by R packages (http://www.r-project.org/).

Finally, to explore the possible reasons underlying this interesting distribution pattern of haplotypes in *SLC30A9*, we performed a correlation between the haplotype frequency of H2 in each population and the corresponding zinc deficiency status. Previous studies have estimated the global prevalence of zinc deficiencies based on zinc availability in national food supplies and the prevalence of stunting^20^. Their preceding results have indicated that inadequate dietary zinc intake may particularly occur in Sub-Saharan Africans and south Asians. For example, about 20%, 11% and 10% of the population have inadequate zinc intake in YRI, CHB and CEU, respectively^20^. Most of the countries in Africa were marked as being in the “HIGH” risk category of zinc deficiency. Here we directly adopted the zinc related data previous researchers used to analyze the relationship between zinc status and populational haplotype distributions (Figure 7A and Table S3). As shown in Figure 7A, populations in those areas with high prevalence of zinc deficiency (YRI and LWK) are more likely to harbor H1 of *SLC30A9*. For instance, in YRI the frequency of H1 was 84.1%, but the zinc deficiency state was 21%, which is the highest among all populations. However, H2 was dominant in the populations with a low prevalence of zinc deficiency. For example, the frequencies of H2 in CEU (76.5%) and CHB (96.4%) were much higher than in YRI (8%), but the zinc deficiency states in CEU (9.6%) and CHB (11%) were lower than in YRI (21%). We then demonstrated a strong correlation between the haplotype frequency of H2 and the zinc deficiency state (R^2^ = 0.5, P = 0.003, Figure 7B). We further included additional 51 populations from Human Genome Diversity Project (HGDP)^29^ in our analysis and also observed strong correlation between H2 and zinc deficiency (R^2^ = 0.38, P = 3.4 × 10^−8^, Figure S12), giving further support to natural selection acting on *SLC30A9*.

## Discussion

With recent developments of high-through DNA genotyping and sequencing technologies, genome-wide scans for genes that have been targeted by selection have become feasible^26^,^28^,^30^,^31^,^32^. These studies screened out several categories of genes that have undergone natural selection, for example, electron transporter genes and peroxisome transporter genes. But it was still necessary to unfold which changes in environment or habit that one category of genes have adapted to. Our work provides such an example that may advance our understanding of human evolution and molecular evolution. In this study, we found that ZTGs have very different population differentiation patterns, both globally and regionally. Some genes have higher global population differentiation levels, such as *SLC30A9* and *SLC30A3*, while *SLC30A6* is more conserved (Figure S1). Moreover, our results showed that ZTGs exhibit significantly larger percentages of genetic variants with high *F_ST_* than random genes do, indicating there are great genetic differentiations in ZTGs among populations (Figure 1A). Generally, several factors can influence population differentiation of a certain gene, such as natural selection, genetic drift and migration^33^,^34^. Under neutral evolution, population differentiation is influenced solely by genetic drift, which increases differentiation versus migration, which decreases differentiation. These two factors, drift and migration, are expected to have the same average effect across the genome. However, natural selection impacts population differentiation only in specific regions^33^,^34^. We ruled out the possibility that drift could cause this differentiation landscape in ZTGs by comparing 24 14ZTGs with random genes (Figure 1B). Despite the marginally significant P-value (0.048) of permutation, we suggested that natural selection had acted on ZTGs. Furthermore, another analysis that indicating nSNPs with high *F_ST_* and low DAF (<0.05) in CEU and CHB are significantly enriched in ZTGs (P = 0.019) (Figure 3C), which has provided more evidence that natural selection events may have occurred on ZTGs.

The analysis above was based on locus-specific *F_ST_*, which revealed patterns of genetic differentiations that are incompatible with neutral expectations in ZTGs among worldwide populations. We then applied two commonly used approaches, iHS and CLR, to detect natural selection in ZTGs; several genes exhibited significant signals (Figure 4). In general, there were selective sweeps in three genes (*SLC30A*8, *SLC30A*9 and *SLC39A*11) that are shared among different continental populations. In principle, haplotypes inherited from ancestral populations might also lead to sharing selective signals between populations. However, this is likely a small effect because recombination for >1000 generations will break down such unusually long haplotypes^30^. Therefore, this might be due to selective events these populations have experienced.

Among these 3 genes, *SLC30A9* was reported to be strongly selected in East Asian populations; it was among the top 10 selective signals in the genome-wide detection of positive selection in human populations^26^,^28^. However, we detected the selective signal in this gene not only in East Asians, but also in Europeans and Africans (Figures 4–5 and S8). This is probably because the footprints that selection left in European populations and African populations were not strong enough to be screened by the genome-wide selection scan in previous studies. Surprisingly, the selective pressure directions in East Asians and Africans were the opposite. It is notable that two functional haplotypes, H1 and H2, are dominant in Africans and East Asians, respectively (Figures 5 and 6). A linear regression was performed to reveal the relationship between the zinc deficiency state and H2 of *SLC30A9* in corresponding populations (Figure 7 and S12). Although the zinc deficiency data used here may not be similar with those of the past and could also be biased due to economic states and dietary habits, these are the best data available so far to investigate the underlying selective force of *SLC30A9*. The significant result of correlation suggests that individuals in continents with low deficiency states are more likely to harbor H2 to perform zinc transporting than those individuals in continents with high deficiency states. We speculated that the zinc transporting ability of *SLC30A*9 with H1 and H2 is different and the local zinc concentration affected frequency of the allele which was favored by natural selection. That is, ancient Africans were forced to adapt to low zinc concentrations, while ancient East Asians and Europeans adapted to the high zinc concentrations so the serum or cell concentration could be maintained properly. Further, preceding investigations proved that zinc concentrations or deficiencies can regulate the transcriptional expression of *SLC30A9* and other ZTGs in many model organisms, such as zebra fish^35^ and rats^36^, supporting our inference that local zinc concentration could be a putative selective force shaping the asymmetric distribution pattern of haplotypes in *SLC30A9*. It is possible that the N-terminal of a transporter protein could affect its transporting ability^37^. A non-synonymous SNP, rs1047626, located in the N-terminal (50th amino acid) of *SLC30A9* (Figure 6C) shows a high *F_ST_* value (0.387) (Table 1). We have speculated that it is the putative causal SNP, resulting in different transporting abilities between H1 and H2.

The correlation (Figure 7) between zinc deficiency and the frequency of H2 in *SLC30A9*, which exhibited a great proportion of high *F_ST_* (Figure S3) and significant selective signals (Figure 4), provides us a good example for investigating the underlying force that results in high differentiations of the ZTGs across different ethnic groups. Many other genes in ZTGs, such as *SLC30A3* and *SLC30A4*, which harbored a great proportion of high *F_ST_* and *SLC30A7*, *SLC30A8* and *SLC39A11*, which showed significant selective signals probably exhibited a similar pattern with *SLC30A9*, because the regulation of serum or cellular zinc concentrations is a complex biological process in which many ZTGs are definitely involved. Therefore, we speculate that some genes in ZTGs were also selected by the uneven continental distribution of absorbable zinc in soils and crops or otherwise in the food chain, since humans spread from Africa and colonized most of the globe. Local adaptation consequently resulted in high differentiations of ZTGs across different ethnic groups. Other evolutionary scenarios cannot be entirely ruled out and should be investigated in detail. One possible scenario is that, in addition to transporting zinc, ZTGs transport other microelements, such as cadmium and manganese^38^, which may also make ZTGs adapt to the environment, leading to high differentiation. What's more, not only does it act as a transporter, *SLC30A9* is located in nucleus and performs as a nuclear receptor co-activator to regulate gene expression^39^. “Nutritional immunity”^40^,^41^ may also explain the evolutionary force. According to the nutritional immunity hypothesis, the human host restricts access to certain micronutrients so that pathogens become less virulent. One recent study used this hypothesis to interpret the selective force of *SLC39A4* in Sub-Saharan Africa^19^. The findings have indicated that the underlying evolutionary force that led to population differentiations of ZTGs still needs to be investigated further.

In summary, proteins coded by Zinc Transporter Genes (ZTGs) play pivotal roles in decreasing or increasing zinc concentrations in cells and thus keep homeostasis of zinc in human body. Genetic variations in ZTGs could directly contribute to population heterogeneity in zinc transporting capabilities. In this study, we outline for the first time the genetic differentiation of worldwide populations and footprints of natural selection upon a comprehensive list of ZTGs. We demonstrated that high differentiations exist in ZTGs among populations. Besides, we identified 17 potentially functional SNPs with allele frequency highly differentiated among populations, which may affect either the protein structures or the expression levels of ZTGs. These results may enhance our understanding of the importance of zinc levels in human evolutionary history and facilitate further functional studies of ZTGs and medical studies on worldwide nutrient problem as well as zinc-related diseases.

## Methods

### Genetic variation data

We analyzed the latest release of the data (version 3 of phase 1, March 2012 release) from the 1000 Genomes Project with autosomal SNPs of 1,092 individuals representing 14 populations worldwide^42^. According to the 1000 Genomes Project Steering Committee, the 14 populations were derived from four ancestries: East Asian ancestry (ASN: CHB, CHS, JPT), African ancestry (AFR: ASW, LWK, YRI), European ancestry (EUR: GBR, FIN, IBS, TSI, CEU) and American ancestry (AMR: CLM, MXL, PUR). The ancestry information of SNPs was obtained from the 1000 Genomes Project (http://ftp.1000genomes.ebi.ac.uk/vol1/ftp/phase1/analysis_results/supporting/ancestral_alignments/).

### Obtaining gene information for zinc transporter genes

Mammalian zinc transporters come from two major families, the *SLC30* (ZnT) family and the *SLC39* (Zip) family^43^. There are 10 14ZnT transporters and 14 14Zip transporters encoded in the human genome. Their genes are designated as *SLC30A1-10* and *SLC39A1-14*, respectively. Gene coordinate information was obtained from the UCSC Table Browser (http://genome.ucsc.edu/cgi-bin/hgTables) to infer the start and end position (hg19) for each gene (Table S4 and Figure S13).

### Population differentiation and F_ST_ estimation

To evaluate whether the genetic variance between the four continental regions is significantly different from the genetic variance among populations within each region, AMOVA was carried out using Arlequin^44^. To investigate global differentiation among ZTGs, we calculated the weighted average *F_ST_* (WA - *F_ST_*) of multiple sites from haplotypes of each gene across all 14 populations. The genetic differentiation between populations of each locus was measured using the unbiased estimates of *F_ST_*, following Weir and Hill^21^ with a python script. We determined empirical cutoffs for the top 1% and 5% of signals genome-wide. Thus, loci or genes with an *F_ST_* value greater than the cutoffs were considered as highly differential SNPs (selected SNPs) or genes. As a result, the highest 1% and 5% of the genome-wide locus-specific *F_ST_* is 0.183 and 0.092 with the average being 0.017.

### Functional SNPs prediction

The functional effects of each SNP from each ZTGs gene were obtained based on the variance effect prediction tools from the Ensembl database^45^. The SNPs that affect gene expression were studied based on the RegulomeDB database^46^. In addition, we studied the SNPs with clear clinical effects and disease-related effects, which were then collected and annotated in the PharmGKB database and the GWAS catalog, respectively^47^,^48^.

### Permutation test

Two kinds of permutation were used in our study. One was used to discern that the *F_ST_* distribution of ZTGs was different from random genes of the whole genome. We randomly sampled 24 unrepeated genes from the whole genome gene list 10,000 times. In every instance, we calculated the proportion of SNPs with *F_ST_* values higher than 0.183. Finally, we obtained 10,000 percentage values representing the whole genome and 1 value (0.0182) for ZTGs; we then sorted these values in descending order. Thus, the index (478 of 10,000) of value for ZTGs represents the P-value of this permutation, which is 0.0478. The distribution of percentage values shows that the ratio (0.0182) of ZTGs is larger than the highest 5% cutoff value (0.0180) of the whole genome (Figure 1B).

The other permutation attempts to examine whether nSNPs with high *F_ST_* (> = 0.092, top 5 percentile of *F_ST_* values) in Africans are enriched in ZTGs, and to exclude the possibility that drift leads to this pattern. We randomly sampled 24 genes from the UCSC database without repetition 10,000 times. Every time, for random genes we calculated the proportion that the number of nSNPs with high DAF in YRI but low DAF (<0.05) in CEU and CHB accounted for the number of SNPs with high *F_ST_*.

### Detecting signals of selection

Two approaches, the integrated haplotype score (iHS) and the composite likelihood ratio (CLR) test, were used to detect the signals of recent positive selection. Because of the hitchhiking effect, positive selection might bring a selected allele into high frequency rapidly enough that recombination does not have time to break down this haplotype, resulting in a long haplotype in high frequency^49^. The iHS test is based on the long haplotype, which is a distinctive signature that could not be expected under neutral drift^30^. It has been shown to possess power enough to identify recent, incomplete sweeps. The standardized iHS scores were calculated for every SNP with minor allele frequency >5% by an R package, rehh^50^. For every gene in each population, we screened the iHS value of each locus and inferred a positive selection signal if there are 7 or more loci with |iHS| equal to or more than the top 5% of genome-wide signals in any continuous 50-SNPs bin of this gene region.

The CLR test, a model-based method, is a statistic to compute the likelihood ratio of selective sweeps by comparing the spatial distribution of allele frequencies in a given window, compared to the frequency spectrum of null distribution, such as all the autosomal regions^51^. In this study, the SweepFinder^52^ program was used to carry out the calculation. For the CLR test, we calculated the standardized CLR score of each population for the entire autosomal regions and took the values with an empirical P-value of 0.05 as the cutoff to detect a natural selection signal at given ZTGs genes.

### Haplotype analysis

To visualize the long haplotype of *SLC30A9*, EHH plot and bifurcation diagrams were drawn using an R package rehh^50^. *SLC30A9* spans about 97 14Kb in the chromosome region 4p13. We defined that *SLC30A9* haplotypes were composed of 7 non-synonymous SNPs (Table S2): rs147121215, rs1047626, rs151273121, rs115329927, rs2581423, rs181235146 and rs141510850. Then, we counted the number of haplotypes composed of these chosen SNPs and computed the corresponding proportion in each population using python script.

### Correlation test

Three indicators of zinc status at the population level has been recommended: (1) the percentage of the population with plasma (serum) zinc concentrations below an appropriate cut-off, (2) the prevalence of usual dietary zinc intakes below the Estimated Average Requirement (EAR), and (3) stunting prevalence^53^,^54^,^55^,^56^. The zinc status data for specific regions or countries was directly downloaded from a published paper that estimated the global prevalence of zinc deficiency^20^. We used a proportion of the population with inadequate zinc intake as an indicator for zinc deficiency and investigated its correlation with H2 (CGGAGAC) of *SLC30A9*, which is strikingly common in East Asians. All significance tests were performed using R packages (http://www.r-project.org/).

### Membrane protein topology and phenotype variation prediction

The transmembrane helices and topology of ZTGs were predicted using HMMTOP^23^ and visualized with TeXtopo^57^. To predict how amino acid variants might change the function of the peptides of ZTGs, Polyphen-2^22^ was used.

## Supplementary Material

Supplementary Information Supplementary Information

## Figures and Tables

**Table 1:** Summary information of highly differentiated functional SNPs at ZTGS. It is noted that in the column “allele”, the first allele is the ancestral state, while the second one with highlight is the derived state. PRD, probably disease-causing; BNG, benign; iTMD, in transmembrane domain; NT, N-terminus; nTMD, near transmembrane domain; IV, intro variant; UGV, upstream gene variant; RRV, regulatory region variant; LS- *F_ST_*, Locus-specific *F_ST_*

rsID	Gene	Position	Allele	F_EUR_	F_ASN_	F_AFR_	F_AMR_	LS-*F_ST_*	Type	Database	Effect or Related		PolyPhen-2	Topology and location
**rs1871534**	*SLC39A*4	8:145639681	G/**C**	0.002	0.000	0.873	0.081	0.763	Missense	RegulomeDB		Leu/Val	PRD: 0.991	iTMD (Fig. S4A)
**rs1047626**	*SLC30A9*	4:42003671	A/**G**	0.750	0.953	0.126	0.737	0.387	Missense	-		Met/Val	BNG: 0.000	NT (Fig. 6C)
**rs2466517**	*SLC39A*11	17:70943990	T/**C**	0.001	0.002	0.343	0.055	0.212	Missense	RegulomeDB		Thr/Ala	BNG: 0.000	nTMD (Fig. S4B)
**rs11011935**	*SLC39A*12	10:18280113	T/**C**	0.000	0.000	0.238	0.022	0.158	Missense	RegulomeDB		Phe/Leu	BNG: 0.000	nTMD (Fig. S4C)
**rs2010519**	*SLC39A*13	11:47431728	G/**A**	0.001	0.012	0.221	0.000	0.135	Missense	-		Glu/Gly	BNG: 0.000	NT (Fig. S4D)
**rs2272662**	*SLC39A*4	8:145639726	T/**C**	0.541	0.496	0.087	0.416	0.130	Missense	RegulomeDB		Thr/Ala	BNG: 0.012	iTMD (Fig. S4A)
**rs75920625**	*SLC39A*4	8:145639654	T/**C**	0.001	0.000	0.135	0.018	0.092	Missense	RegulomeDB		Thr/Ala	BNG: 0.012	nTMD (Fig. S4A)
**rs61756712**	*SLC30A4*	15:45814306	A/**G**	0.001	0.008	0.342	0.045	0.224	Splice	RegulomeDB		-	-	
**rs759071**	*SLC39A*3	19:2728577	G/**A**	0.327	0.632	0.817	0.283	0.168	eQTL	RegulomeDB		-	-	
**rs6832846**	*SLC39A*8	4:103181749	A/**G**	0.787	0.526	0.986	0.705	0.112	eQTL	RegulomeDB		-	-	
**rs151368**	*SLC39A*8	4:103181113	A/**T**	0.787	0.526	0.986	0.705	0.112	eQTL	RegulomeDB		-	-	
**rs11889699**	*SLC39A*10	2:196528101	A/**G**	0.588	0.366	0.127	0.377	0.108	eQTL	RegulomeDB		-	-	
**rs17278473**	*SLC39A*8	4:103243101	C/**T**	0.400	0.024	0.179	0.324	0.097	eQTL	RegulomeDB		-	-	
**rs151372**	*SLC39A*8	4:103174196	C/**T**	0.787	0.528	0.951	0.700	0.093	eQTL	RegulomeDB		-	-	
**rs950027**	*SLC30A4*	15:45801035	T/**C**	0.452	0.947	0.953	0.652	0.210	IV	GWAS		Response to fenofibrate	-	
**rs11264736**	*SLC39A*1	1:153939130	T/**C**	0.494	0.949	0.730	0.666	0.123	UGV	GWAS		Lentiform nucleus volume	-	
**rs17060812**	*SLC39A*14	8:22228828	C/**T**	0.039	0.066	0.341	0.070	0.111	RRV	PharmGKB		Nortriptyline	-	
