# Supplementary material for: Differential Natural Selection of Human Zinc Transporter Genes between African and Non-African Populations
Source: Sci Rep. 2015 Apr 30;5:9658. doi: 10.1038/srep09658 (PMC5386188; doi:10.1038/srep09658)
Supplement: Supplementary Information [file srep09658-s1.pdf]

Differential Natural Selection of Human Zinc Transporter Genes between African and Non-African Populations

Running title: **Natural Selection of Human Zinc Transporter Genes**

Chao Zhang,[1,§] Jing Li,[1,§] Lei Tian,[1] Dongsheng Lu,[1] Kai Yuan,[1] Yuan Yuan,[1] Shuhua Xu[1,*]

[1]Max Planck Independent Research Group on Population Genomics, Chinese Academy of Sciences and Max Planck Society (CAS-MPG) Partner Institute for Computational Biology (PICB), Shanghai Institutes for Biological Sciences, Chinese Academy of Sciences, Shanghai 200031, China.

[§] These authors contributed equally to this work.

* To whom correspondence and requests for materials should be addressed.

xushua@picb.ac.cn (S.X.)

Supplementary information:

Table S1. Genetic differentiation analysis including AMOVA, WA-F_{ST} and a high locus-specific F_{ST} proportion of ZTGs. The cutoff values of empirical distribution of the whole genome are given.

Table S2. Frequencies of haplotypes composed of 7 nSNPs of *SLC30A9* in 14 populations.

Table S3. Estimated country-specific prevalence of zinc deficiency. We used the mean percentage of the population with inadequate zinc intake from 1990-2005 as an indicator to perform a correlation with the haplotype frequency of H2. Note that the zinc deficiency state of PUR is from the Dominican Republic because of the missing statistical data for PUR.

Table S4. Information of 24 ZTGs, including start and end positions (hg19) in corresponding chromosome.

Figure S1. AMOVA of ZTGs among populations. The higher dashed line represents the 95% cutoff value of the empirical distribution of all genes from the genome. Another dashed line indicates the mean values of all genes. *SLC30A9* and *SLC30A3* have greater variance among groups, while *SLC30A6* has a low variance, indicating that this gene maybe functionally conserved.

Figure S2. Bar plot of WA-F_{ST} of ZTGs. The higher and lower dashed lines represent the 95% and 5% cutoff values of empirical distribution of all genes from the genome, respectively. *SLC30A9* and *SLC30A3* have a high WA-F_{ST}, while *SLC30A6* has a low value.

Figure S3. Bar plot showing the proportion of numbers of SNPs with F_{ST} >0.092 in each gene. The higher dashed line represents the 95% cutoff value of empirical distribution of all genes from the genome. Another dashed line indicates the mean values of all genes.

Figure S4. Predicted membrane topology of ZTGs generated using HMMTOP and visualized with TeXtopo. (A) Topology of *SLC39A4*. (B) Topology of *SLC39A11*. (C) Topology of *SLC39A12*. (D) Topology of *SLC39A13*. Arrows indicate locations of nSNPs with high F_{ST}.

Figure S5. Worldwide derived allele frequency distributions for (A) rs2466517, (B) rs11011935, (C) rs2010519, (D) rs75920625 and (E) rs2272662. World maps here were created by R packages (http://www.r-project.org/).

Figure S6. Selective signatures for *SLC30A7*.

Figure S7. Selective signatures for *SLC30A8*.

Figure S8. Selective signatures for *SLC30A9*.

Figure S9. Selective signatures for *SLC9A11*.

Figure S10. Overlapping selected SNPs with iHS value >= cutoff. (A) Venn diagram of selected SNPs in CEU, CHB and YRI. (B) Allele state of selected SNPs with high iHS values. About 3 (of 8) SNPs overlap between CEU and CHB; all of them were selected at ancestral alleles. However, only 2 (of 55) SNPs overlap between CHB and YRI. Interestingly, derived alleles of rs4362859 and rs9990477 were favored in CHB, while the ancestral alleles were selected in YRI.

Figure S11. Network for *SLC30A9* haplogroups composed of 7 nSNPs. H1 and H2 are different at rs1047626.

Figure S12. The correlation between the frequency of rs1047626 in 51 populations from HGDP and zinc deficiency state of the corresponding populations.

Figure S13. Locations of ZTGs at the chromosomes.

Figure S1. AMOVA of ZTGs among populations.

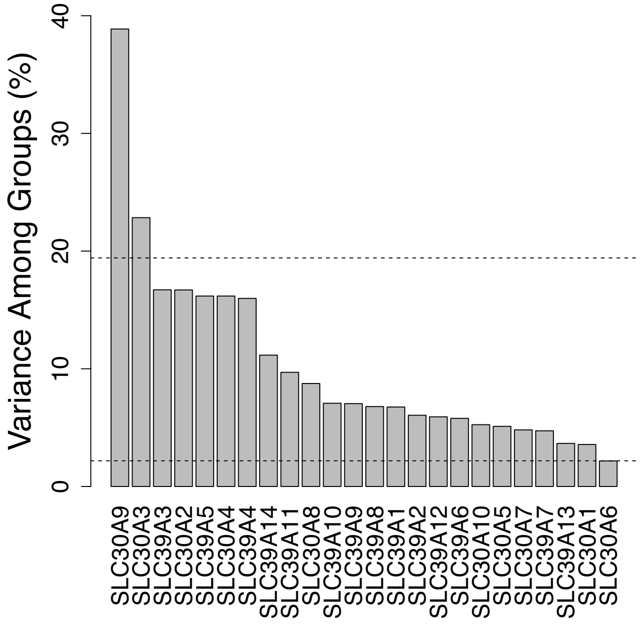

Figure S2. Bar plot of WA-F_{ST} of ZTGs.

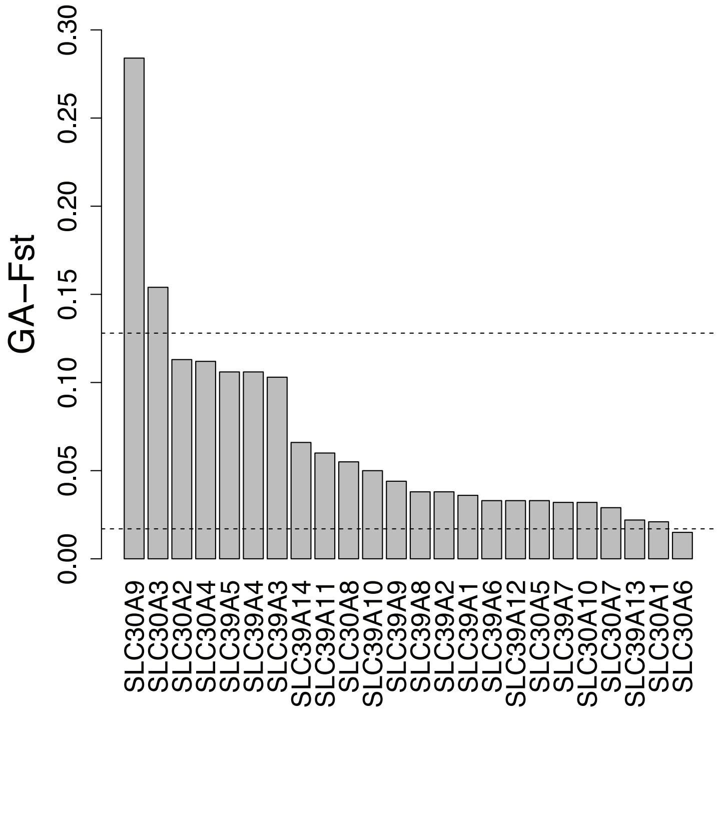

Figure S3. Bar plot showing the proportion of numbers of SNPs with F_{ST} >0.092 in each gene.

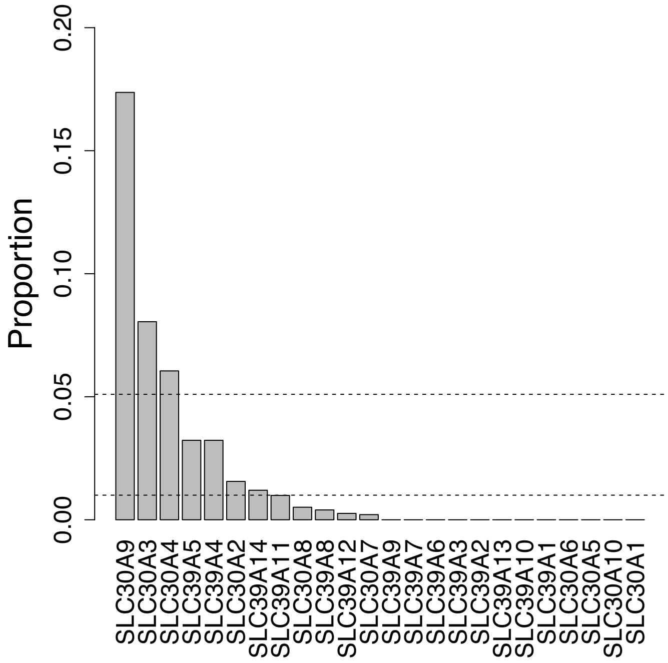

Figure S4. Predicted membrane topology of ZTGs generated using HMMTOP and visualized with TeXtopo.

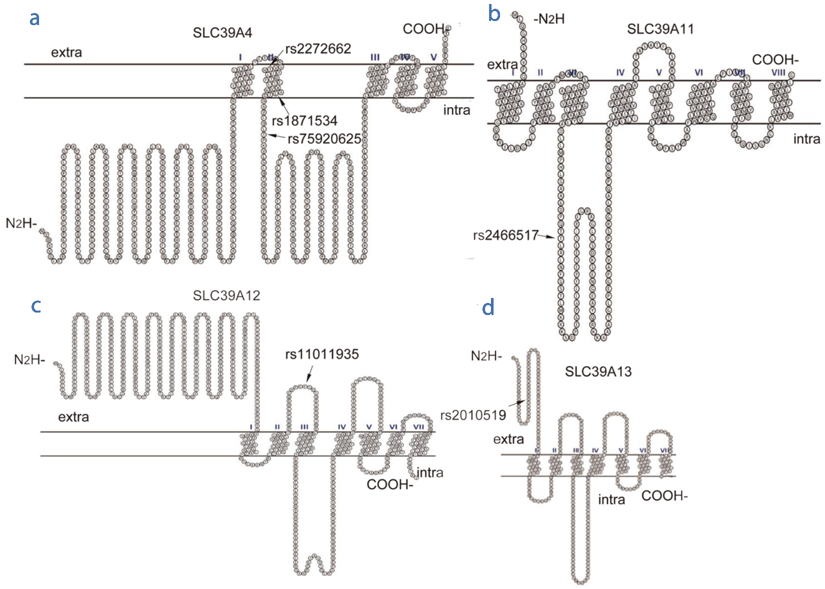

Figure S5. Worldwide derived allele frequency distributions for (A) rs2466517, (B) rs11011935, (C) rs2010519, (D) rs75920625 and (E) rs2272662. World maps here were created by R packages (http://www.r-project.org/).

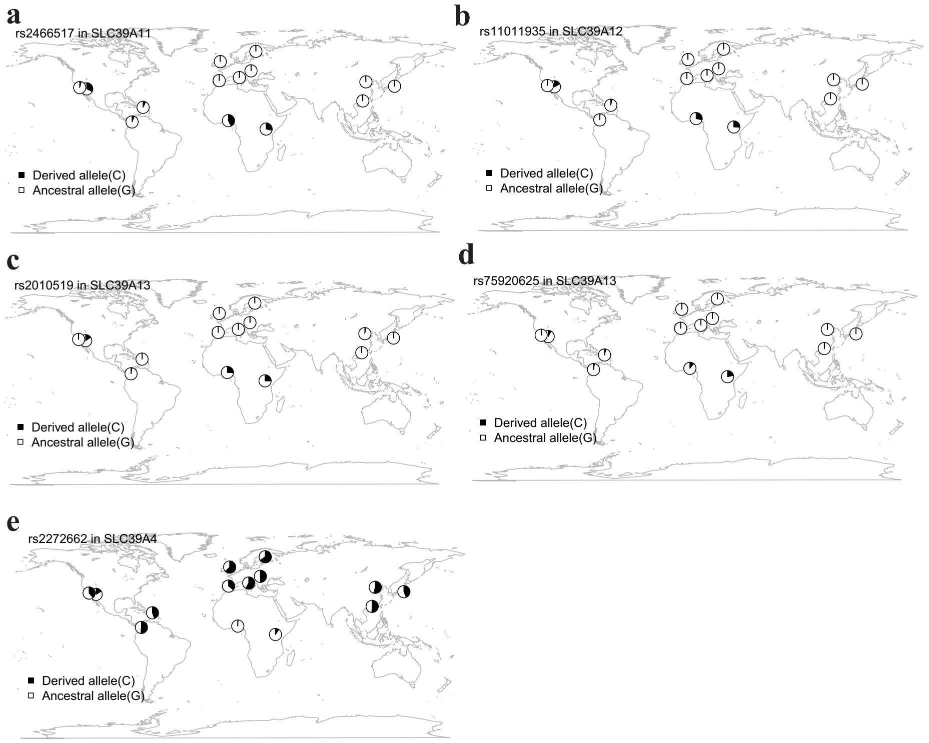

Figure S6. Selective signatures for *SLC30A7*.

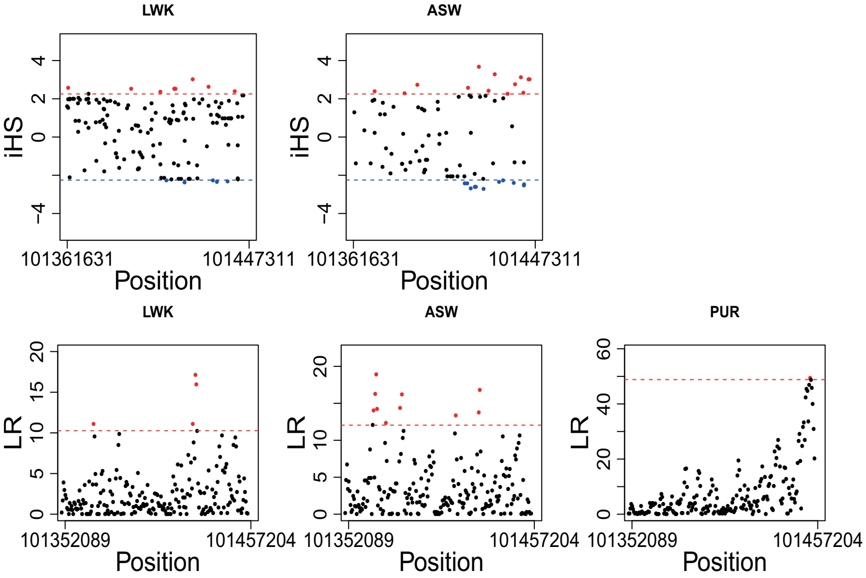

Figure S7. Selective signatures for *SLC30A8*.

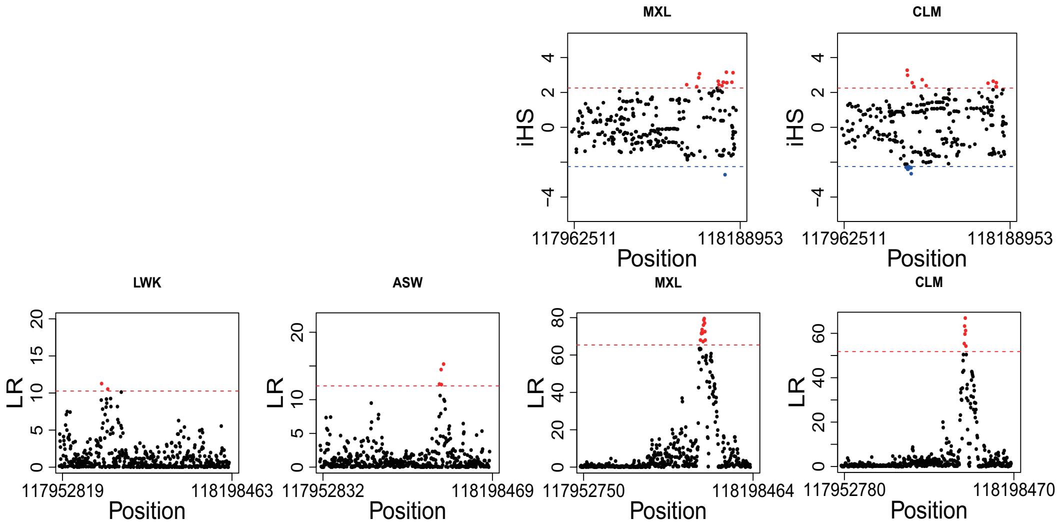

Figure S8. Selective signatures for *SLC30A9*.

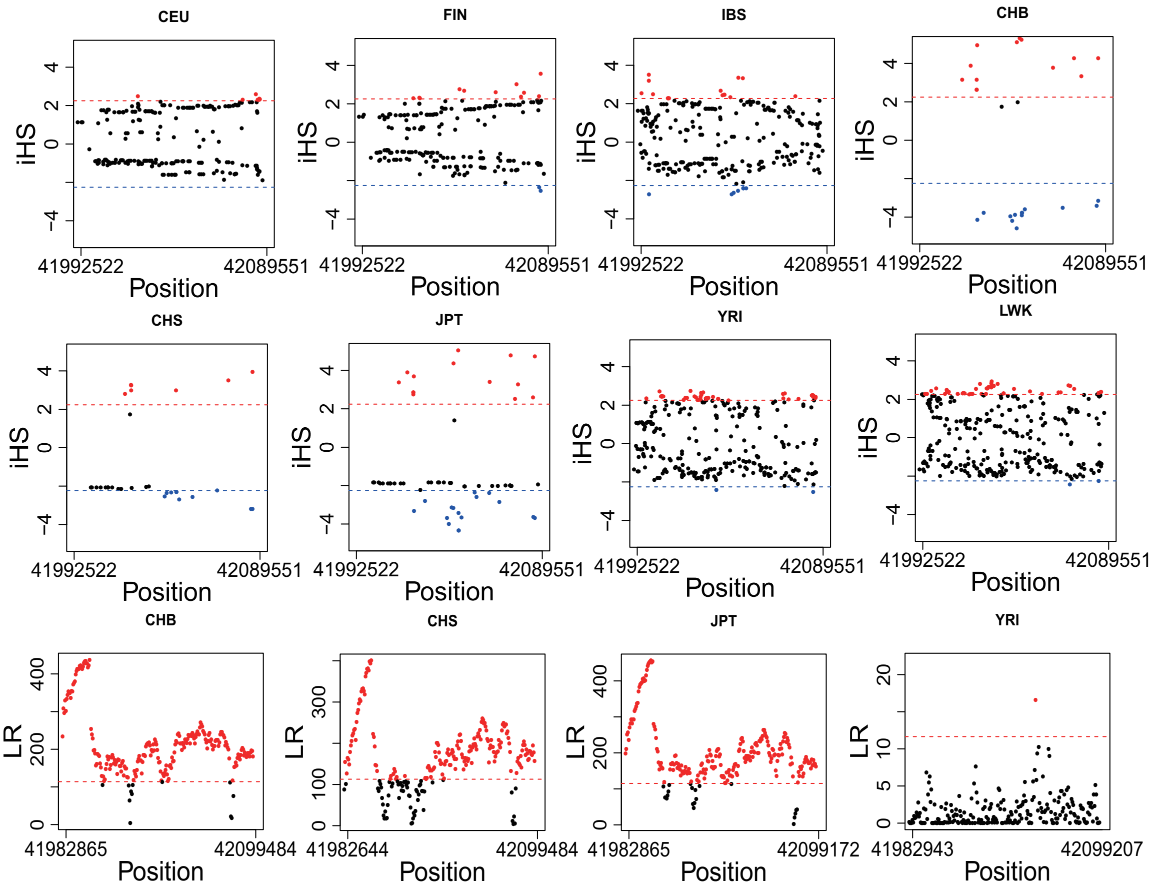

Figure S9. Selective signatures for *SLC9A11*.

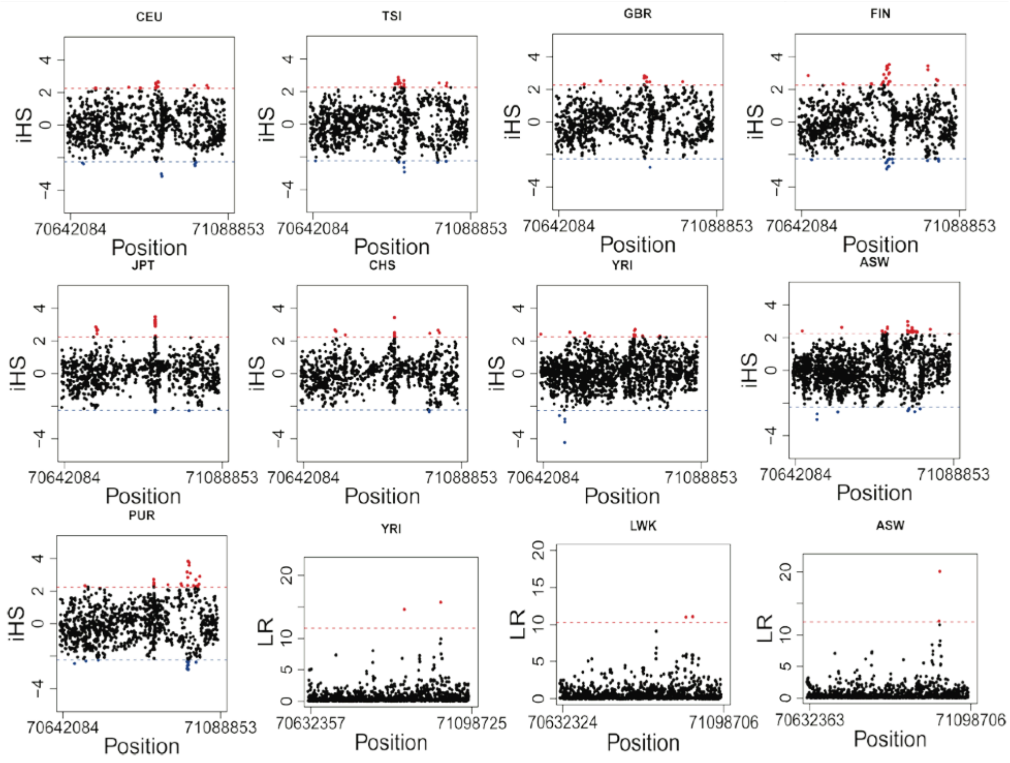

Figure S10. Overlapping selected SNPs with iHS value >= cutoff.

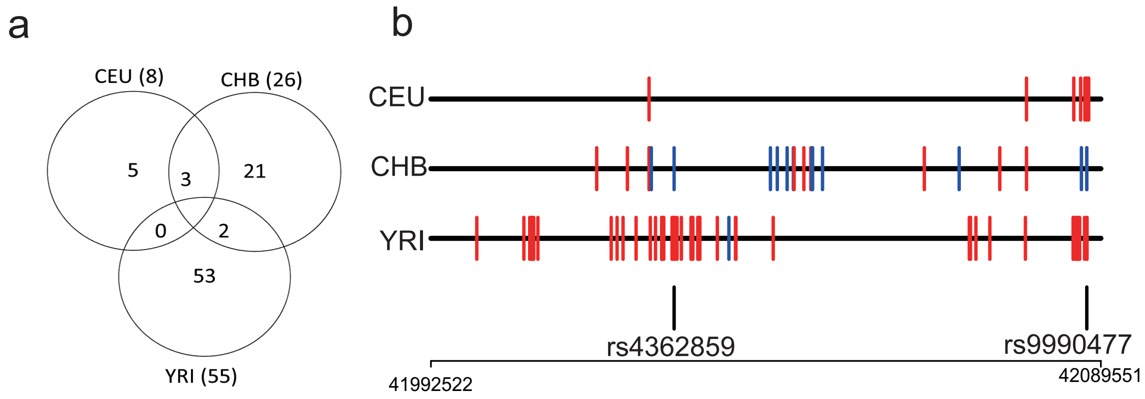

Figure S11. Network for *SLC30A9* haplogroups composed of 7 nSNPs. H1 and H2 are different at rs1047626.

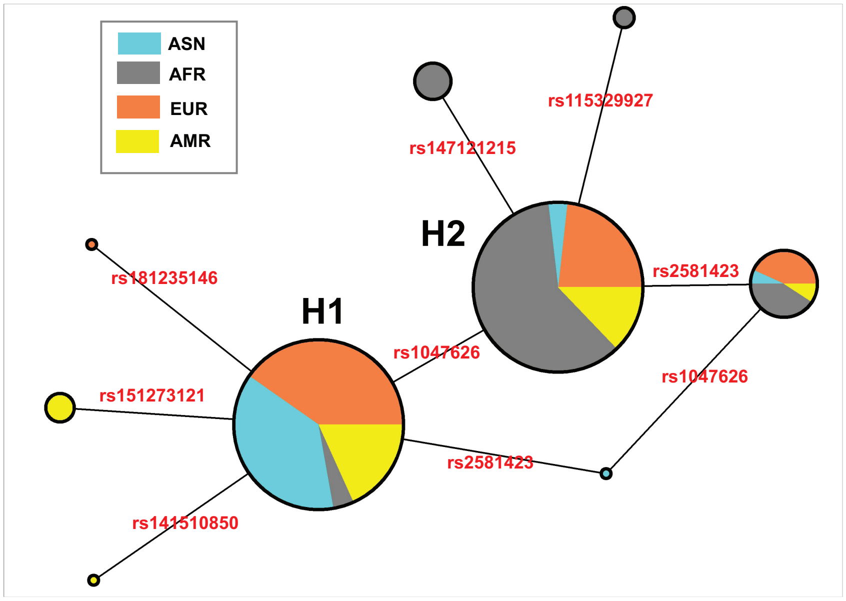

Figure S12. The correlation between the frequency of rs1047626 in 51 populations from HGDP and zinc deficiency state of the corresponding populations.

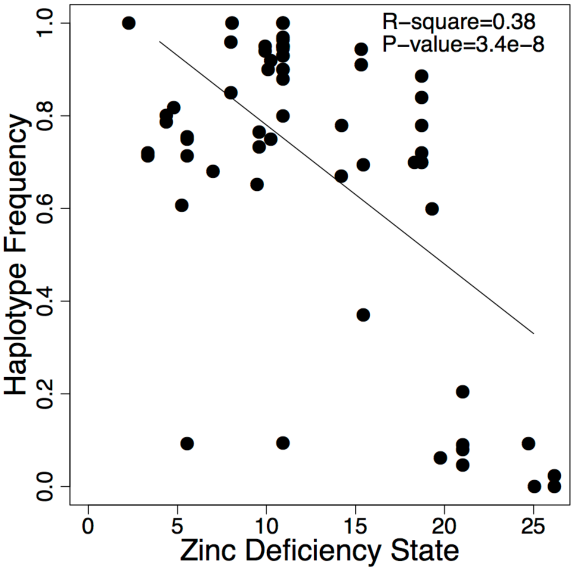

Figure S13. Locations of ZTGs at the chromosomes.

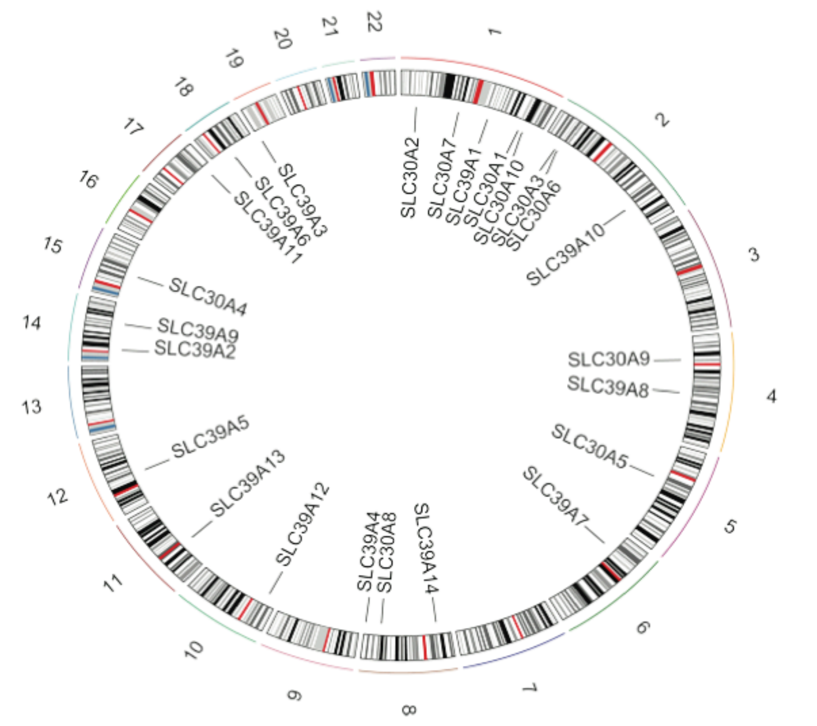

Table S1. Genetic differentiation analysis including AMOVA, WA-F_{ST} and a high locus-specific F_{ST} proportion of ZTGs. The cutoff values of empirical distribution of the whole genome are given.

	Among groups	Among populations within groups	Within populations	GA-Fst	porp(>=top 1%)
**SLC30A1**	3.57	0.88	95.55	0.021	0
**SLC30A2**	16.77	1.33	81.97	0.113	0
**SLC30A3**	22.84	0.88	76.28	0.154	0.081
**SLC30A4**	16.18	1.58	82.24	0.112	0.061
**SLC30A5**	5.11	1.18	93.71	0.033	0
**SLC30A6**	2.17	1.2	96.64	0.015	0
**SLC30A7**	4.81	0.91	94.28	0.029	0.002
**SLC30A8**	8.75	0.85	90.4	0.055	0.005
**SLC30A9**	38.87	1.1	60.03	0.284	0.174
**SLC30A10**	5.25	0.83	93.92	0.032	0
**SLC39A1**	6.75	0.21	93.05	0.036	0
**SLC39A2**	6.05	0.97	92.97	0.038	0
**SLC39A3**	16.71	0.16	83.13	0.103	0
**SLC39A4**	15.98	1.08	82.93	0.106	0.032
**SLC39A5**	16.18	0.91	82.91	0.106	0.032
**SLC39A6**	5.79	0.62	93.59	0.033	0
**SLC39A7**	4.73	1.32	93.95	0.032	0
**SLC39A8**	6.79	0.39	92.82	0.038	0.004
**SLC39A9**	7.04	0.88	92.09	0.044	0
**SLC39A10**	7.07	1.71	91.22	0.05	0
**SLC39A11**	9.07	0.68	89.62	0.06	0.01
**SLC39A12**	5.92	0.42	93.66	0.033	0.003
**SLC39A13**	3.66	0.96	95.39	0.022	0
**SLC39A14**	11.16	0.25	88.59	0.066	0.012
**Mean of ZTGs**	10.32	0.89	88.79		
**Mean of whole genome**	9.36	0.69	89.94		
**Top 1% of whole genome**	27.53	2.07	99.81		
**Top 5% of whole genome**	19.42	2.07	97.13	0.128	0.051
**Low 1% of whole genome**	0.07	-0.16	71.44		
**Low 5% of whole genome**	2.18	0.13	79.83	0.017	0.01

Table S2. Frequencies of haplotypes composed of 7 nSNPs of *SLC30A9* in 14 populations.

Haplotype: **rs147121215-rs1047626-rs151273121-rs115329927-rs2581423-rs181235146-rs141510850**

**haplo**	CEU	TSI	GBR	FIN	IBS	CHB	JPT	CHS	YRI	LWK	ASW	MXL	PUR	CLM	Total
**CGGAAAC**	0.000	0.000	0.000	0.000	0.000	0.000	0.006	0.000	0.000	0.000	0.000	0.000	0.000	0.000	0

**CAGAGAC(H1)**	0.200	0.240	0.197	0.156	0.321	0.031	0.039	0.055	0.841	0.825	0.754	0.136	0.373	0.217	0.304
**CGGAGGC**	0.000	0.000	0.000	0.000	0.036	0.000	0.000	0.000	0.000	0.000	0.000	0.000	0.000	0.000	0
**TAGAGAC**	0.000	0.000	0.000	0.000	0.000	0.000	0.000	0.000	0.023	0.046	0.000	0.000	0.000	0.000	0.006
**CGGAGAC(H2)**	0.765	0.755	0.787	0.801	0.607	0.964	0.944	0.945	0.080	0.093	0.205	0.818	0.600	0.750	0.663
**CGGAGAT**	0.000	0.000	0.000	0.000	0.000	0.000	0.000	0.000	0.000	0.000	0.000	0.000	0.009	0.000	0
**CGAAGAC**	0.000	0.000	0.000	0.000	0.000	0.000	0.000	0.000	0.000	0.000	0.000	0.038	0.009	0.017	0.004
**CAGTGAC**	0.000	0.000	0.000	0.000	0.000	0.000	0.000	0.000	0.011	0.000	0.016	0.000	0.000	0.000	0.002
**CAGAAAC**	0.035	0.005	0.017	0.043	0.036	0.005	0.011	0.000	0.045	0.036	0.025	0.008	0.009	0.017	0.02

Table S3. Estimated country-specific prevalence of zinc deficiency. We used the mean percentage of the population with inadequate zinc intake from 1990-2005 as an indicator to perform a correlation with the haplotype frequency of H2. Note that the zinc deficiency state of PUR is from the Dominican Republic because of the missing statistical data for PUR.

Population	Zinc Deficiency State	H2 Frequency
**YRI**	21.025	0.08
**LWK**	24.725	0.093
**CHB**	10.925	0.964
**CHS**	10.925	0.945
**JPT**	15.325	0.944
**ASW**	21.025	0.205
**MXL**	4.8	0.818
**CLM**	10.25	0.75
**PUR**	19.3	0.6
**CEU**	9.6	0.765
**FIN**	4.375	0.801
**IBS**	5.25	0.607
**TSI**	5.55	0.755
**GBR**	4.375	0.787

Table S4. Information of 24 ZTGs, including start and end positions (hg19) in corresponding chromosome.

gene_symbol	chrom	strand	txStart	txEnd	length

**SLC30A1**	chr1	-	211748380	211752099	3719
**SLC30A2**	chr1	-	26364513	26372604	8091
**SLC30A3**	chr2	-	27477439	27485960	8521
**SLC30A4**	chr15	-	45774679	45815002	40323
**SLC30A5**	chr5	+	68389775	68426899	37124
**SLC30A6**	chr2	+	32390909	32449181	58272
**SLC30A7**	chr1	+	101361631	101447311	85680
**SLC30A8**	chr8	+	117962511	118188953	226442
**SLC30A9**	chr4	+	41992522	42089551	97029
**SLC30A10**	chr1	-	220087605	220101993	14388
**SLC39A1**	chr1	-	153931574	153935844	4270
**SLC39A2**	chr14	+	21467413	21470034	2621
**SLC39A3**	chr19	-	2734523	2740074	5551
**SLC39A4**	chr8	-	145637797	145642273	4476
**SLC39A5**	chr12	+	56623819	56631629	7810
**SLC39A6**	chr18	-	33688493	33709357	20864
**SLC39A7**	chr6	+	33168602	33172214	3612
**SLC39A8**	chr4	-	103182820	103266348	83528
**SLC39A9**	chr14	+	69865384	69929107	63723
**SLC39A10**	chr2	+	196521851	196602426	80575
**SLC39A11**	chr17	-	70642084	71088853	446769
**SLC39A12**	chr10	+	18240767	18332221	91454
**SLC39A13**	chr11	+	47430045	47438051	8006
**SLC39A14**	chr8	+	22224761	22291640	66879
